# Tripartite Interactions of *Barley Yellow Dwarf Virus*, *Sitobion avenae* and Wheat Varieties

**DOI:** 10.1371/journal.pone.0106639

**Published:** 2014-09-03

**Authors:** Xiao-Feng Liu, Xiang-Shun Hu, Mike A. Keller, Hui-Yan Zhao, Yun-Feng Wu, Tong-Xian Liu

**Affiliations:** 1 State Key Laboratory for Crop Stress Biology in Arid Areas, Northwest A&F University, Yangling, Shaanxi, China; 2 Key Laboratory of Crop Pest Management on the Northwest Loess Plateau, Ministry of Agriculture, Northwest A&F University, Yangling, Shaanxi, China; 3 School of Agriculture, Food and Wine, Waite Campus, the University of Adelaide, Adelaide, Australia; Swedish University of Agricultural Sciences, Sweden

## Abstract

The tripartite interactions in a pathosystem involving wheat (*Triticum aestivum* L.), the *Barley yellow dwarf virus* (BYDV), and the BYDV vector aphid *Sitobion avenae* were studied under field conditions to determine the impact of these interactions on aphid populations, virus pathology and grain yield. Wheat varietal resistance to BYDV and aphids varied among the three wheat varieties studied over two consecutive years. The results demonstrated that (1) aphid peak number (APN) in the aphid + BYDV (viruliferous aphid) treatment was greater and occurred earlier than that in the non-viruliferous aphid treatment. The APN and the area under the curve of population dynamics (AUC) on a *S. avenae*-resistant variety 98-10-30 was significantly lower than on two aphid-susceptible varieties Tam200(13)G and Xiaoyan6. (2) The production of alatae (PA) was greater on the variety 98-10-30 than on the other varieties, and PA was greater in the aphid + BYDV treatment on 98-10-30 than in the non-viruliferous aphid treatment, but this trend was reversed on Tam200(13)G and Xiaoyan6. (3) The BYDV disease incidence (DIC) on the variety 98-10-30 was greater than that on the other two varieties in 2012, and the disease index (DID) on Tam200(13)G was lower than on the other varieties in the aphid + BYDV and BYDV treatments in 2012, but not in 2011 when aphid vector numbers were generally lower. (4) Yield loss in the aphid + BYDV treatment tended to be greater than that in the aphid or BYDV alone treatments across varieties and years. We suggested that aphid population development and BYDV transmission tend to promote each other under field conditions. The aphids + BYDV treatment caused greater yield reductions than non-viruliferous aphids or virus treatment. Wheat varietal resistance in 98-10-30 affects the aphid dispersal, virus transmission and wheat yield loss though inhibits aphid populations from increasing.

## Introduction

Tripartite interactions among crop host, plant virus and insect vector are more complicated than that we have imaged. The most devastating viruses of crop plants around the world are vectored, often obligately, by hemipterous insects among which aphids, whiteflies and leafhoppers predominate [1]–[3]. Some insect vectors are also important pests of crop plants. The host plant, vector and virus are always interdependent components of a complex pathosystem that co-exists within the field ecosystem [4]. The host plant is sessile within such a plant pathosystem, however, the plant characteristics (e.g. growth, reproduction, emitted volatiles and plant nutrients) can be altered substantially by pathogen infection. The primary traits of insect vectors, such as fecundity, survival, and behavior are altered in response to altered host plant characteristics [4]–[13]. These changes in the host plant and insect vector could affect the spread of the insect pests and the viruses they carry. From a practical perspective, a change in the status of virus and its vectors is expected to affect the yield of the host crop. Plants can have various levels of resistance to an insect vector, which can increase pest mortality, extend the development time of nymphs, and decrease offspring production [14]. Unfortunately, at present we do not know how such resistance characters in plants that influence the population dynamics of viruses, insect vectors and the overall tripartite interactions in the field.

Wheat, *Triticum aestivum* (L.), is one of the most important crops in the world, and the aphid *Sitobion avenae* (F.) is an important worldwide pest of wheat. *S. avenae* has a strong preference to feeding on the rachis and base of the spikelets of the wheat head. It can lead to yield component changes including reducing the number of wheat heads per plant, grains per wheat head and grain weight. *Barley yellow dwarf viruses* (BYDVs) are phloem-limited *Luteoviruses* that infect most members of the Poaceae family including wheat, barley (*Hordeum vulgare* L.) and oat (*Avena sativa* L.). The viruses interfere with plant physiological processes, which cause dwarfing, chlorosis, stunting, and yield loss [15]–[17].

The typical population development of *S. avenae* includes an initial slow build-up, rapid multiplication, slow down, stagnation, and a final rapid decrease. In northern China, the initial slow build-up phase of *S. avenae* population occurs from mid-March to mid-April, the rapid multiplication phase occurs from the end-April to the mid-May, the slow down and stagnation phases occur at the end of May, and the rapid decrease occurs at the beginning to the mid-June [18]–[20]. Aphid population proliferation in early- and mid-May can cause direct yield loss [19]. *S. avenae* occurs almost every year in central Shaanxi Province, China [20].

Chinese BYDV isolates have been classified into four species according to Rochow's system, namely GAV, PAV, GPV and RMV. BYDV-GAV is most efficiently transmitted by *Schizaphis graminum* (Rondani) and *S. avenae*. Efficient insect vectors of BYDV-PAV, BYDV-GPV and BYDV-RMV are *S. avenae* and *Rhopalosiphum padi* L., *S. graminum* and *R. padi*, and *R. maidis* (F.), respectively [21], [22].

The acquisition of BYDV can directly alter host selection behavior of its insect vector. For example, after acquiring BYDV, *R. padi* prefers feeding on non-infected wheat plants, while non-infective aphid prefers BYDV-infected plants [23]. This evolution of strategies in plant pathogens enhances their spread to new hosts. BYDV also could indirectly alter vector insect behavior through inducing wheat plant biochemical changes. Wheat plants infected BYDV have reduced chlorophyll content and lower rates of photosynthesis [16], as well as increased total amino acid content [24], which improved the nutritional quality of wheat leaf for aphids. These biochemical changes in wheat affect the development, fecundity, even field population dynamics of the vector [25]–[27]. BYDV infection reduces the host plant's suitability in terms of aphid population growth potential, but aphids produced more alatae on the virus-infected spring wheat [28]. More alatae enhance the spread of the virus in the field and thus largely influence the epidemiology of both the pest vector and disease. Previously researchers have studied tripartite interactions in this host-vector-virus pathosystem often under laboratory conditions [24]–[28], and the nature of the interactions has not been validated under field conditions.

Under field conditions, the yield reductions caused by aphids and/or BYDV infection varies greatly in different years and in different regions. Some factors, such as the viral isolates, time of infection, varietal resistance to aphid vector and/or virus, planting date and water stress have been identified to affect yield reduction at a given aphid density or virus incidence [29]–[34]. Most previous investigations focused on yield losses induced by non-viruliferous aphid infestation or BYDV infection alone [29]–[52]. Few studies were focused on the yield losses caused by both aphid infestation and viral infection under field conditions [53]–[54].

The wheat-BYDV-*S. avenae* pathosystem provides an ideal model to investigate tripartite interactions. It has been reported that *S. avenae* plays an important vector role in spreading of the BYDV-PAV from winter hosts to wheat, barley, and maize in the spring in France [55]. Although *S. graminum* is an important insect vector of the BYDV in some regions [56], [57], *S. avenae* is a more prominent aphid species in northwestern China, where it plays an key role as a vector that spreads of this virus [18], [43]–[45], [55]–[58], the proportion of *S. avenae* carrying BYDV-GAV, which is a prominent BYDV species in China, ranged from 2.5–91.5% in wheat fields [59]. Wheat varietal resistance is an especially advantageous trait to suppress aphids [60], as it is an efficient and environmentally friendly way to controlling them [61]. However, few studies have reported the effects of varietal resistance on wheat yield losses caused by both *S. avenae* infestation and BYDV infection together. In this study, we used the wheat-*S. avenae*-BYDV pathosystem to explore tripartite interactions among aphid population dynamics, visual pathogenicity, and yield responses of wheat varieties that vary in their resistance to aphids and BYDV. Plant was infested by non-viruliferous *S. avenae* alone, infected by BYDV alone, or jointly infested by viruliferous *S. avenae* carrying BYDV under field conditions. The results have clear implications for the broader understanding of tripartite interactions of plant-virus-vector pathosystems, including the epidemiology of insect-transmitted plant viruses, the role of varietal resistance in wheat in natural settings.

## Methods and Materials

We state clearly that no specific permissions were required for these locations/activities. We confirm that the field studies did not involve endangered or protected species.

### Plants, BYDV and Aphids

The cultivar Xiaoyan6, a hybrid offspring of *T. sativum* and *Agropyrum repens* Beauvois ( = *T. repens*), was chosen because it was widely grown in northern China with better quality, higher yield and more stress tolerance than other varieties. However, it is susceptible to *S. avenae* and BYDV [62]. The variety 98-10-30 was chosen due to its resistance to *S. avenae* [14] and susceptibility to BYDV. The variety Tam200(13)G was chosen for its tolerance to BYDV and susceptibility to *S. avenae* (XSH unpublished data).

The viruliferous *S. graminum* with BYDV were originally obtained from an infected wheat field in Yongshou County, Shaanxi Province, China. The BYDV species was identified as a GAV using RT-PCR [59], [63]. Viruliferous *S. graminum* were transferred to clean wheat seedling (variety Batis, non-infested by BYDV and non-infested by aphids) in a segregated cage at 25±0.5°C (day), 22±0.5°C (night) with a 16 h light: 8 h dark photoperiod, and approximately 70±10% R.H. When the wheat seedlings displayed obvious chlorosis symptoms, the aphids were cleaned using a brush, and BYDV-infected wheat seedlings were obtained. The non-viruliferous *S. avenae* were originally collected in a wheat field, Yangling, China, and was confirmed as BYDV-free using multiplex RT-PCR [63]. The *S. avenae* were inoculated on BYDV-infected wheat seedlings for one week to obtain viruliferous aphids with BYDV-GAV.

The viruliferous and non-viruliferous *S. avenae* were separately maintained on clean Batis seedlings in separate cages under growth chamber conditions under the same conditions as described above for one year before the experiments. The wheat seedlings were replaced about once a month. The aphids were examined regularly using RT-PCR to ensure that the populations remain non-viruliferous or BYDV-GAV infected, respectively [59], [63].

### Field experiments

The field experiments were performed in winter wheat experimental fields at Northwest A&F University (central Shaanxi Province, China, north latitude 34° 17′ 35″, east longitude 108° 4′ 18″). In the first year, the wheat was sown on Oct. 15, 2010, and harvested on June 5, 2011; in the second year, the wheat was sown on Oct. 11, 2011, harvested on June 6, 2012. The kernel weight of each seed lot was determined prior to planting so that all varieties were sown at the same rate (300 kernels m^−2^). Prior to planting, nitrogen in the form of ammonium nitrate at 10 kg/666.7 m^2^ was applied to the experimental plots. An additional 10 kg/666.7 m^2^ of nitrogen was top-dressed in stem elongation stage of winter wheat in March.

The field plots were arranged in a split-plot design with three wheat varieties, Xiaoyan6, 98-10-30 and Tam200(13)G, as main plot; and four aphid-BYDV treatments as subplots, five repeats (blocks) for each treatment (Appendix S1: A). The area of repeat block was 20 m^2^, and the blocks were 4 m long and consisted of 20 rows spaced 25 cm apart. The four aphid-BYDV treatments included a non-viruliferous aphid treatment, a BYDV treatment (virus infection alone), a combined aphid + BYDV treatment (viruliferous *S. avenae* infestation), and a control (without aphid infestation and virus infection) (Appendix S1: B). In the BYDV treatment, 240 viruliferous aphids (5 adult aphids per 100 wheat plants) were uniformly released to a 12 m^2^ (3 m×4 m) area in the center of each block (the same below). Three days later, imidacloprid (2.5% WP 8000×, 20 g a.i./hm^2^. Shijiazhuang Yaoyuan Pharmaceutical Technology Co., Ltd., Shijiazhuang, Hebei, China) was sprayed to kill the aphids. The control plots were also sprayed with imidacloprid to eliminate any possibility of aphid infestation. In the aphid treatment, 240 non-viruliferous adult aphids (5 aphids per 100 wheat heads) were uniformly released; and in combined aphid + BYDV treatment, 240 viruliferous aphids (5 adult aphids per 100 wheat heads) were uniformly released. We were released all aphids on March 26, 2011, and March 24, 2012. Imidacloprid was applied again because the aphid number increased to 100 per 100 wheat heads in some blocks sampled of BYDV treated and control on May 6, 2012.

### Data Collection and Analysis

Considering the dispersal of aphids among neighboring plots, all data collection was performed on a 12 m^2^ (3 m×4 m) area in the center of each repeated block. Aphids in each block were counted once a week after aphid infestation. Five points were sampled per block, and 20 wheat heads per point each time. Wheat head density, numbers of apterae and alatae, and visual BYDV symptoms ratings were recorded in filling stage (May 14, 2011, and May 12, 2012). And then the five points in one block were added together (100 heads per block).

The aphid peak number (APN, max aphid number) and the area under the curve of population dynamics (AUC) were integrated to estimate the aphid population development. The production of alatae (PA  =  alatae/total adults) was determined. A BYDV rating from 0 to 10 was assigned according to the standard of classification (Appendix S2) [64].

The BYDV disease incidence (DIC) and disease index (DID) was calculated (DIC  =  symptomatic wheat heads/total wheat heads; DID  = 100× [∑ (disease ratings × the number of that rating)/(total wheat heads ×10)] [64].

At plant maturity, when the stem, leaves and wheat heads had dried, the grain yield and the yield components were measured. Five points were sampled and 30 wheat heads per point were recorded in each block (3 m×4 m area in the center). The yield components included the number of kernels per wheat head (HK) and the weight of 1,000 kernels (KW). The actual grain yield (AY) was measured. As different wheat varieties have specific yield characters, the AY, HK and KW loss ratios were calculated using the following formula: loss ratio  =  (control value - actual value)/control value.

The parameters, APN, AUC, PA, DID, DIC, and the loss ratios of HK, KW and AY were analyzed using split-plot design ANOVA (DPS software) [65]. APN was transformed using natural logarithm transformation; and PA, DIC, the loss ratios of HK, KW and AY were transformed using arcsine transformation to reduce the variance value. The means were separated by Tukey's test under α = 0.05. All figures were drawn with SigmaPlot 12.1 (Systat Software Inc., Chicago, IL, USA).

The full names of all acronyms in this paper were listed in Appendix S3.

## Results

### *S. avenae* population dynamics on three wheat varieties under aphid-BYDV treatments

**Figure 1:**
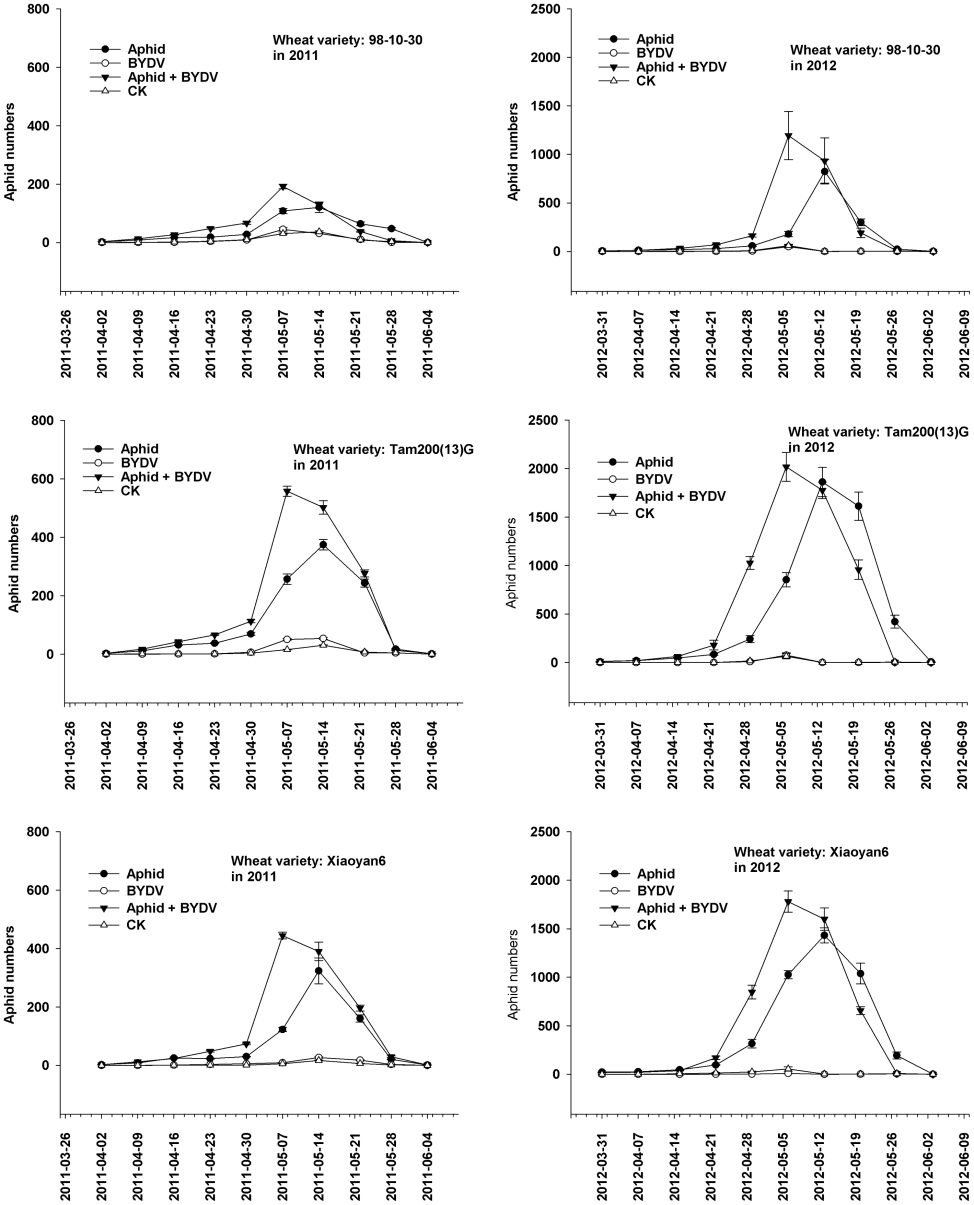
The population dynamics of *S. avenae* on three wheat varieties treated with *S. avenae* infestation, BYDV infection alone or jointly (mean ± SD).

Population of *S. avenae* displayed consistent patterns of abundance according to the four treatments between years and among the three wheat varieties (Fig. 1). The aphid peak number (APN) occurred in the first half of May each year. The aphid peak number occurred one week earlier when viruliferous aphids and BYDV (aphid + BYDV treatment) were combined than when non-viruliferous aphids alone (aphid treatment) were present. Negligible numbers of aphids were found on plants in the control and BYDV treatments at the time of peak numbers, as aphid were suppressed before they could significantly affected the results.

**Figure 2:**
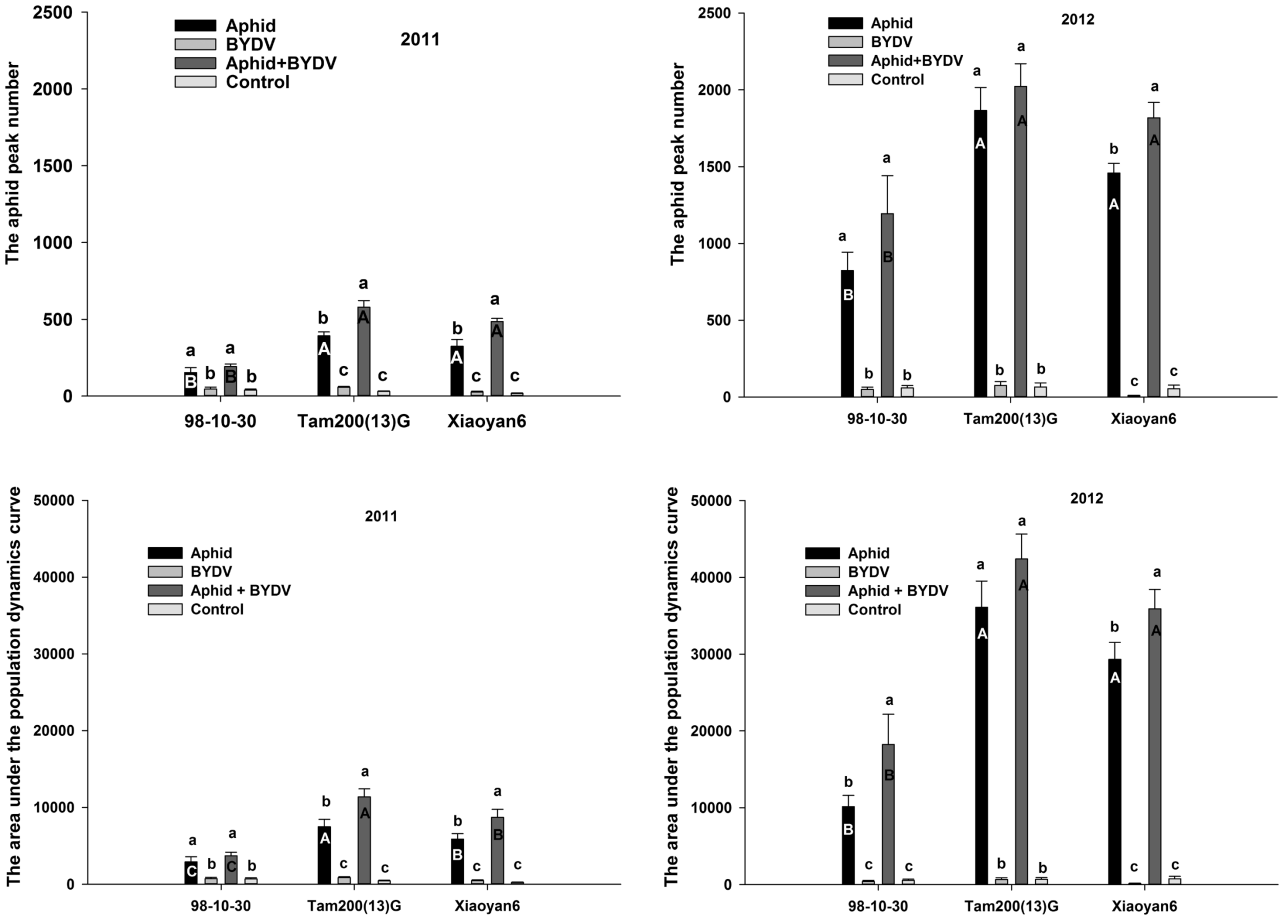
The aphid peak number (APN) and the area under the curve of population dynamics (AUC) on three wheat varieties treated with *S. avenae* infestation, BYDV infection alone or jointly (mean ± SD). Note: APN and AUC both were significantly different among three wheat varieties (2011: p_APN_ = 0.006, p_AUC_<0.001; 2012: p_APN_ = 0.014, p_AUC_<0.001); among four treatments (p_APN_ and p_AUC_ all less than 0.001 in both years); and interactions between varieties and treatments (2011: p_APN_ = 0.001, p_AUC_<0.001; 2012: p_APN_ = 0.001, p_AUC_<0.001). Different little letters above the bar indicate the significance of differences according to treatment for same variety. Different capital letters in the bar indicate the significance of differences according to varieties for same treatment (p<0.05).

Patterns of aphid pressure, as indicated by the aphid peak numbers (APN) and the area under the curve of population dynamics (AUC) were largely consistent according to treatment, variety and year of study (Fig. 2). The averages both APN and AUC were lower on the resistant variety 98-10-30 than on the susceptible varieties Tam200(13)G and Xiaoyan6 in both years (*p*<0.05, the same as in the following). While the APNs were not significantly different between Tam200(13)G and Xiaoyan6 in both years. The AUCs were not significantly different between Tam200(13)G and Xiaoyan6 in 2012, but different in 2011.

Among the subplots (aphid and/or BYDV treatments), the averages of both APN and AUC in the combined aphid + BYDV treatments were significantly greater than those in the non-viruliferous aphids treatments.

The APN and AUC differences between treatments for each of three varieties in both years are presented in Fig. 2. On wheat variety 98-10-30, the APNs were not significantly different between aphid + BYDV treatment and non-viruliferous aphid treatment in both years. However, the AUC in the aphid + BYDV treatment was significantly greater than that in the non-viruliferous aphid treatment in 2012. On Tam200(13)G, the APN and AUC of aphid + BYDV treatment were significantly greater than that in the non-viruliferous aphid treatment in 2011, but not in 2012. On Xiaoyan6, the APN and AUC of non-viruliferous aphid treatment were obviously lower than that of the aphid + BYDV treatment in both years.

**Figure 3:**
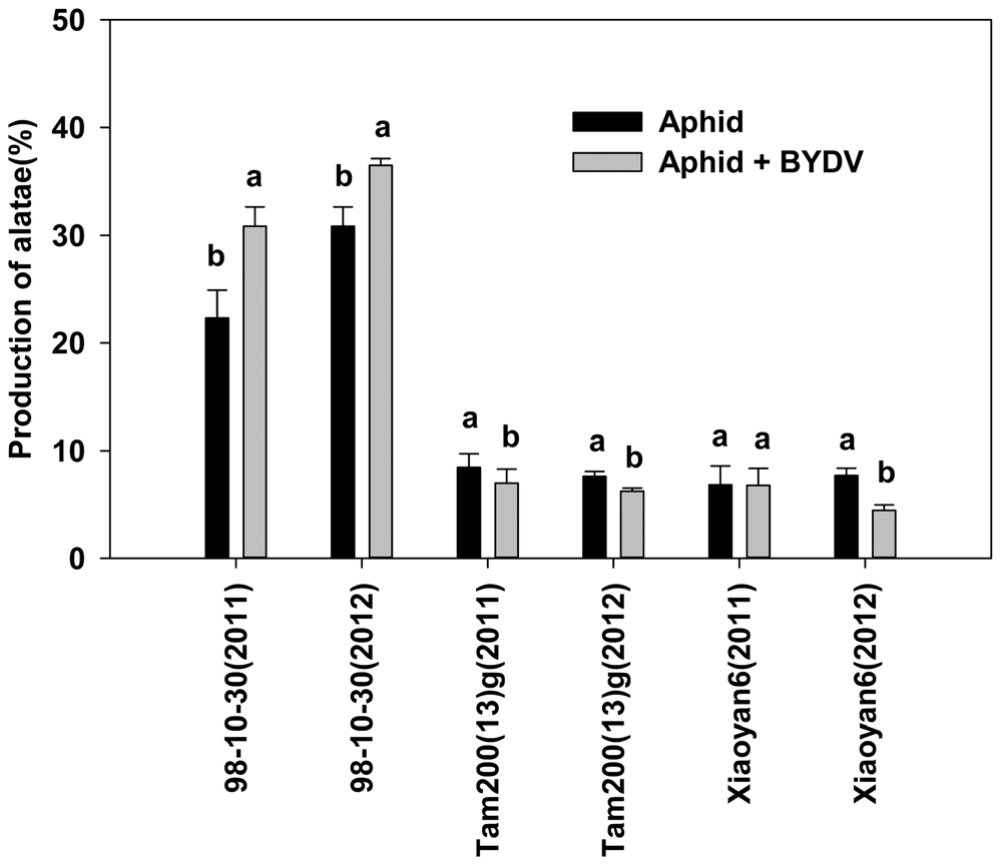
Production of alataes on three wheat varieties infested with non-viruliferous *S. avenae* or viruliferous *S. avenae* (mean ± SD). Note: Different letters above the bar indicate the significance of differences according to treatment for same variety (p<0.05).

The production of alatae on 98-10-30 was significantly greater than that on Tam200(13)G and Xiaoyan6. The aphid + BYDV treatment produced 30.8±4.4% alatae in 2011, and 36.5±0.6% alatae in 2012, which were obviously greater than that the non-viruliferous aphid treatment in both years (22.3±2.6% and 30.8±1.8%, respectively) on resistant 98-10-30. However, this phenomenon was not observed on Tam200(13)G and Xiaoyan6 in both years (Fig. 3).

### BYDV morbidity on three wheat varieties under aphid-BYDV treatments

**Figure 4:**
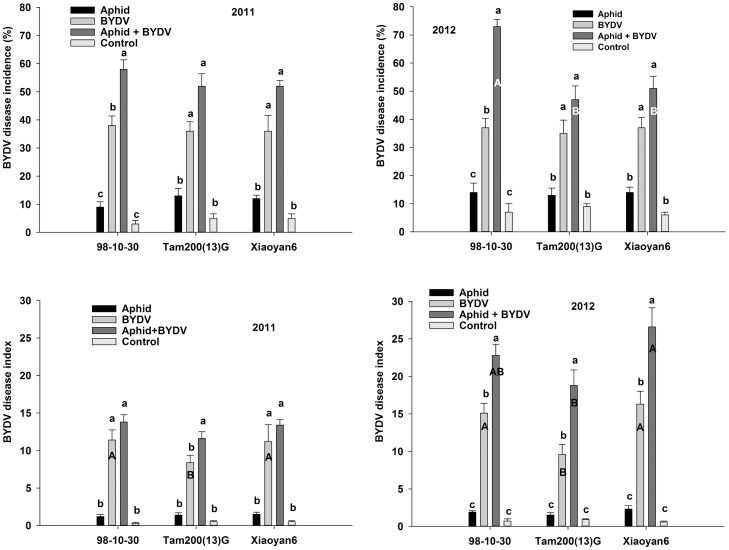
The BYDV disease incidence (DIC) and disease index (DID) of three wheat varieties infected with *S. avenae*, BYDV alone or jointly (mean ± SD). Note: The differences of BYDV DIC were significantly among treatments (2011: p<0.001; 2012: p<0.001); among wheat varieties in 2012 (p = 0.012), not in 2011 (p = 0.887). The interactions between treatment and variety were significant in 2012 (p = 0.002), not in 2011 (p = 0.678). The differences of DID were significant among three wheat varieties (2011: p = 0.007; 2012: p<0.001); among different treatments in both years (2011: p<0.001; 2012: p<0.001); and the interactions between treatment and variety in 2012 (p = 0.023), were not significant interactions between treatment and variety in 2011(p = 0.153). Different little letters above the bar indicate the significance of differences according to treatment for same variety. Different capital letters in the bar indicate the significance of differences according to varieties for same treatment.

The differences of DIC according to the four treatments between years and among the three varieties are shown in Fig. 4. On two *S. avenae*-susceptible wheat varieties, the DICs in the aphid + BYDV treatment were similar to that in the BYDV treatment in two years. But on the *S. avenae*-resistant wheat variety 98-10-30, the DICs in the aphid + BYDV treatment were significantly higher than that in the BYDV treatment in two years. Of the three varieties, the DIC on 98-10-30 was greatest in aphid + BYDV treatment; and it was significantly greater than those on Xiaoyan6 and Tam200(13)G in 2012.

The differences of DID according to the four treatments between years and among the three varieties are presented in Fig. 4. The DIDs of the BYDV treatment were significantly lower than those of the aphid + BYDV treatment on Tam200(13)G in 2011 and on all three wheat varieties in 2012. The DIDs were similar between aphid + BYDV treatment and BYDV treatment on 98-10-30 and Xiaoyan6 in 2011. The DID on Xiaoyan6 was significantly higher than that on Tam200(13)G in the aphid + BYDV treatment in 2012. The DIDs on Xiaoyan6 and 98-10-30 were significantly higher than on Tam200(13)G in the BYDV treatment in both years.

### Wheat yield components response of three wheat varieties to the aphid-BYDV treatments

**Figure 5:**
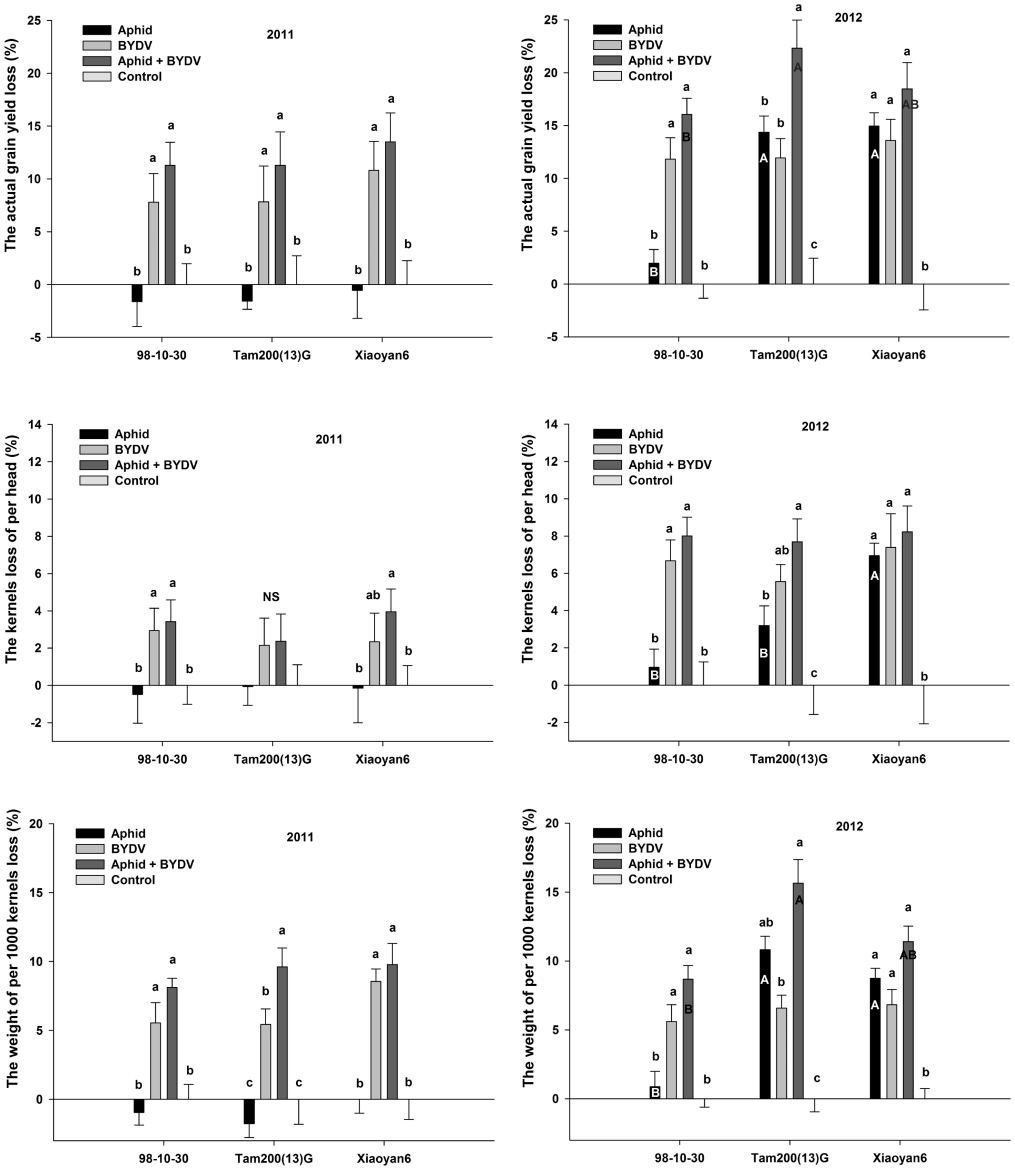
The responses of the actual yield (AY) and yield components, kernels per wheat head (HK) and weight of 1,000 kernels (KW) of three wheat varieties to infected with *S. avenae*, BYDV or both (mean ± SD). Note: AY losses were significantly different among different aphid-BYDV treatments (2011: p<0.001; 2012: p<0.001); among wheat varieties in 2012 (p = 0.048), but not in 2011 (p = 0.598). Two factor interactions were not significantly (2011: p = 0.998; 2012: p = 0.086) (Fig. 5 above). HK losses were significant among treatments (2011: p = 0.017; 2012: p<0.001); were not significant among wheat varieties (2011: p = 0.869; 2012: p = 0.142); and interactions between treatment and variety (2011: p = 0.975; 2012: p = 0.338). KW losses were different among treatments in both years (2011: p<0.001; 2012: p<0.001); among wheat varieties in 2012 (p = 0.012), but not in 2011 (p = 0.564). The two factor interactions were significant in 2012 (p = 0.007), not in 2011 (p = 0.978). Different little letters above the bar indicate the significance of differences according to treatment for same variety. Different capital letters in the bar indicate the significance of differences according to varieties for same treatment. NS is not significant different.

The yield losses of three wheat varieties according four treatments in both years are presented in Fig. 5. In 2011, the non-viruliferous aphid infestation did not induce significantly yield loss compared with the control, but the BYDV and aphid + BYDV treatment induced significantly heavier yield losses on all three wheat varieties; the yield losses were not significantly different between the BYDV treatment and the aphid + BYDV treatment. In 2012, for 98-10-30, BYDV treatment and aphid + BYDV treatment caused significantly higher yield loss, but non-viruliferous aphid infestation did not. For Tam200(13)G, BYDV, non-viruliferous aphid, and aphid + BYDV treatments all caused obviously serious yield loss, the yield loss in the aphid + BYDV treatment was higher than that in the non-viruliferous aphids treatment and the BYDV treatment. For Xiaoyan6, the yield losses in BYDV, non-viruliferous and aphid + BYDV treatments were not significantly different. They were greater than those in the control.

In 2011, the HK and KW loss ratios were similar between the BYDV and the aphid + BYDV treatments, both higher than the non-viruliferous aphid treatment and the control on 98-10-30 and Xiaoyan22. The HK loss ratios were not significantly different among all aphid-BYDV treatments, but the KW loss ratios in the aphid + BYDV treatment was higher than that in the BYDV treatment on Tam200(13)G. In 2012, the trends of HK and KW loss ratios were similar to AY losses on 98-10-30 and Xiaoyan6. The HK loss ratio in aphid + BYDV treatment was obviously higher than that in the non-viruliferous aphid treatment, but the KW loss ratio in the aphid + BYDV treatment was significantly higher than that in the BYDV treatment on the Tam200(13)G.

## Discussion

Virus infected plants had either positive or negative effects on phytophagous vector insects that feeding on the plants [4]–[13], [16], [21]–[28], [66]–[71]. The biology and behavior changes of host plant or vector insect that induce by plant virus infections and/or vector insect infestation have the potential to enhance vector insect fitness and virus transmission [5]–[6], [16], [23], [24]. For example, the persistently transmitted (PT) viruses tend to improve host quality for vectors and promote them for long-term feeding, because the PT viruses need vectors to feed on an infected host for a sustained period to acquire and circulate (and sometimes replicate) virions [4]. *Luteoviruses* (viruses in the family Luteoviridae), including BYDV, are persistently transmitted viruses [72]. In this study, we found that *S. avenae* infestation enhanced the BYDV transmission and severity; and BYDV infection also accelerated the increase of *S. avenae* population, which was in agreement with that Fiebig et al. had reported [28]. The infection with BYDV might induce physiological changes in wheat cultivars which are sensitive to the virus, and hence improve their acceptability for *S. avenae* [71].

It takes less than a minute for *S. avenae* to acquire and transmit BYDV-GAV [34], indicating that *S. avenae* has high transmission efficiency. In our study, the viruliferous aphids had a shorter period of transmission on the wheat plants in the BYDV treatment (aphids admitted three days feeding) than in the aphid + BYDV treatment (aphids admitted continuous feeding). This means that the aphids could produce more viruliferous offspring in the aphid + BYDV treatment than in the BYDV treatment. Therefore, the plants should have greater BYDV DICs or DIDs in the aphid + BYDV treatments than that in the BYDV treatments. In fact, our results showed the DICs were obviously different between aphid + BYDV treatment and BYDV treatment on the *S. avenae*-resistant wheat variety 98-10-30, but did not on the two *S. avenae*-susceptible wheat varieties in both years. We found higher DIDs in the aphids + BYDV treatment than in the BYDV treatment on three wheat varieties in 2012. In addition, the DIDs on Tam200(13)G were lower than that on Xiaoyan6 under BYDV infection in both years and under aphid + BYDV infestation in 2012, but the APNs, AUCs and DICs were not significantly different between Tam200(13)G and Xiaoyan6. These results indicated that the characteristics of host varieties played an important role in BYDV spread and development: a longer duration of viral transmission by the viruliferous aphids increased the disease severity (higher DID) on all wheat varieties; and accelerated the BYDV transmission (higher DIC) on the aphid-resistant variety 98-10-30, but did not on the other two aphids-susceptible wheat varieties. Tam200(13)G (lower DID, higher DIC) could limited BYDV symptom development, but it could not prevent the spread of BYDV. It probably caused by the feeding behavior difference of aphid on the host varieties with different characteristics [71].

The production of alatae (PA) was increased when *S. avenae* and *R. padi* reared on oats infected with BYDV [66]. In this study, we observed that the PA did not increase on two aphid-susceptible varieties in both years, but higher DIC was accompanied with higher PA on aphid-resistant 98-10-30 in aphid + BYDV treatment in both years. It seems that the increase of alatae induced BYDV DIC increase, though aphid peak number (APN) and the area under the curve of population dynamics (AUC) were the lowest on the resistant variety 98-10-30. These results indicated that the *S. avenae*-resistant variety 98-10-30 could prevent *S. avenae* population from developing; however, it increased alatae production and accelerated BYDV transmission. It implicated that planting 98-10-30, a resistant variety to a vector, poses a potential risk of virus transmitting and spreading widely.

The yield losses caused by *S. avenae* (or redeem economic losses through pesticide application to control *S. avenae*) could reach approximately 6–60% in New Zealand [35]; 10% to 30% or higher in Germany [34], [35]; less than 12% and 12–17% in the Great Britain [38]–[40]; 2–63% in China [42]–[45], [59]; 9–30% in Canada [41]. Yield losses caused by BYDV were estimated at 7–58% in the US [31]–[33]; 5–10% in England [46], up to 25% in New Zealand [47], and 7–80% in Canada [48]. In the state of Victoria, Australia, inoculation of BYDV before tillering lowered grain yields by 9–79%, but inoculation of the virus at early stem extension lowered only by 6–9% [49]. Similarly, grain yield could be lowered by 63% in the fall infection and by 41% in the spring infection in Illinois, the US [50]. Why was the yield losses range caused by *S. avenae* or BYDV so wide? Generally, the effect factors including the weather characteristics, wheat varieties, aphid population density, time of infestation, and BYDV incidence and index in different countries and regions. However, a significant, but overlooked, factor was the complex combination of aphid infestation and BYDV infection in the field. Riedell et al. (1999) reported that 21% grain yield was reduced by the *R. padi* treatment, 46% by the BYDV treatment, and 58% by the combination of *R. padi* + BYDV treatment on four winter wheat varieties under laboratory conditions which were absent from additional environmental stresses [53]. In the 6/6 cases (three varieties in two years) of this study, the aphid + BYDV treatments shows higher grain loss than the other treatments. The binomial probability of this result or one more extreme is p = 0.031, suggesting that the aphids + BYDV treatment caused greater yield losses than the aphid or BYDV treatment alone in the field, which was in agreement with that reported by Riedell et al. [53].

Generally, *S. avenae* does not cause damage when they population abundance under 4 aphids/tiller [29]. Hence, the economic thresholds always have been suggested to be 4–5 aphids/head around the flowering stage of the wheat in China [59] and in England [51], and 3–5 aphids/head in Germany [52]. However, the economic thresholds of *S. avenae* have been considered unreliable due to the low or poor correlation between *S. avenae* abundance and yield loss [73]. In fact, the aphid and BYDV population development varied on different wheat varieties. Therefore, it induced different yield losses [31], [74]. The yield loss of a susceptible wheat cultivar “Abe” infected with BYDV-PAV was more severe with increasing numbers of both *R. padi* and *S. avenae* from 5 to 15 pairs/head [54]. In this study, for the variety 98-10-30, we did not observe significantly yield losses in the non-viruliferous aphid treatment compared with the control in both years. However, for the variety Xiaoyan6, it displayed similar yield losses in the three aphid-BYDV treatments compared with the control in 2012. For the variety Tam200(13)G, a significantly higher yield loss was found in the BYDV + aphid treatment than those in the BYDV treatment or aphids treatment alone in 2012 (Fig. 5). Our results in here are consistent with those aforementioned reports [31], [54], [74]. In contrast, these results are not in agreement with that yield reductions caused by BYDV were not significantly different among the winter wheat varieties [33], [75], [76].

Our results did not show yield loss in aphid infected plots in 2011, but displayed significantly heavier yield loss in 2012. The possible reasons were that the APN was less than 400 per 100 wheat heads in 2011 and was more than 800 per 100 wheat heads in 2012 in the non-viruliferous aphid treatment. The historical meteorological data showed that there were more than six dates of moderate rain with wind velocity at 3–4 grade during April and May in 2011, almost once in every ten days; in contrast, there were no such heavy rains and strong wind in 2012. In addition, the dates with low temperature were more in 2011 than in 2012. It appears that rain, wind and low temperature played significant roles in inhibiting the development of aphid population in 2011.

In conclusion, we suggested that *S. avenae* infestation promoted BYDV transmission and spreading, and BYDV infection increased the *S. avenae* population development to some extent under field conditions. Either non-viruliferous aphid infestation or BYDV infection alone or the combination of aphid + BYDV significantly reduced wheat yield. In general, the yield loss was heavier induced by viruliferous aphid (aphid + BYDV treatment) infestation than by non-viruliferous aphid infestation or BYDV infection alone. The resistant characteristics of wheat variety affect the yield loss that caused by BYDV-aphid treatments. Meanwhile, the environmental factors cannot be ignored.

The BYDV-PAV isolates from different countries show great divergences both in genomic sequences and in pathogenicity [77]. It is not yet clear whether the yield responses to different BYDV species are similar. Thus, more advanced quantitative analyses are needed to estimate the interactions of aphids, BYDV species and wheat in the field in future.

## Supporting Information

Appendix S1 **Summary of experimental treatments.** A. Wheat variety susceptibility profiles. B. Split plot treatments applied to each wheat variety.
(DOCX)

Appendix S2 **BYDV disease rating.**
(DOCX)

Appendix S3 **Acronyms.**
(DOCX)
