# Supplementary material for: Tripartite Interactions of Barley Yellow Dwarf Virus, Sitobion avenae and Wheat Varieties
Source: PLoS One. 2014 Sep 3;9(9):e106639. doi: 10.1371/journal.pone.0106639 (PMC4153664; doi:10.1371/journal.pone.0106639)
Supplement: Appendix S1 — Summary of experimental treatments. A. Wheat variety susceptibility profiles. B. Split plot treatments applied to each wheat variety. (DOCX) [file pone.0106639.s001.docx]

A.

|  |  | *Barley Yellow Dwarf Virus* (BYDV) | |
| --- | --- | --- | --- |
|  |  | Susceptible | Tolerant |
| Aphids (*S. avenae*) | Susceptible | Xiaoyan6 | Tam200(13)G |
|  | Resistant | 98-10-30 | - |

B

| Treatments | Aphid suppression with imidicloprid |
| --- | --- |
| Aphid (Non-viruliferous *S. avenae*) | No |
| BYDV | Yes |
| Aphid + BYDV ( viruliferous *S. avenae*) | No |
| Control | Yes |
