# Supplementary material for: Tripartite Interactions of Barley Yellow Dwarf Virus, Sitobion avenae and Wheat Varieties
Source: PLoS One. 2014 Sep 3;9(9):e106639. doi: 10.1371/journal.pone.0106639 (PMC4153664; doi:10.1371/journal.pone.0106639)
Supplement: Appendix S2 — BYDV disease rating. (DOCX) [file pone.0106639.s002.docx]

0 = No symptoms.

1 = Leaf apices chlorosis in the lower canopy.

2 = One leaf chlorosis below flag leaf.

3 = Two leaves chlorosis below flag leaf.

4 = Quarter of flag leaf chlorosis, one leaf chlorosis below flag leaf.

5 = Quarter of flag leaf chlorosis, two leaves chlorosis below flag leaf.

6 = Flag leaf chlorosis.

7 = Flag leaf chlorosis, one leaf chlorosis below flag leaf.

8 = Flag leaf and two leaves chlorosis below flag leaf.

9 = Plants stunting, but heading.

10 = Stunting obviously, and the lack of heading.
