# Supplementary material for: Tripartite Interactions of Barley Yellow Dwarf Virus, Sitobion avenae and Wheat Varieties
Source: PLoS One. 2014 Sep 3;9(9):e106639. doi: 10.1371/journal.pone.0106639 (PMC4153664; doi:10.1371/journal.pone.0106639)
Supplement: Appendix S3 — Acronyms. (DOCX) [file pone.0106639.s003.docx]

| Acronyms | Full name |
| --- | --- |
| BYDV | *Barley yellow dwarf virus* |
| APN | The aphid peak number (max aphid number) |
| AUC | The area under the curve of population dynamics |
| PA | The production of alatae (= alatae / total adults)) |
| DIC | Disease incidence |
| DID | Disease index |
| HK | The number of kernels per wheat head |
| KW | The weight of 1,000 kernels |
| AY | The actual grain yield |
| PT | Persistently transmitted |
